# A cytogenetic study of three parasitic wasp species (Hymenoptera, Chalcidoidea, Eulophidae, Trichogrammatidae) from Brazil using chromosome morphometrics and base-specific fluorochrome staining

**DOI:** 10.3897/CompCytogen.v11i1.11706

**Published:** 2017-03-17

**Authors:** Vladimir E. Gokhman, Fabricio Fagundes Pereira, Marco Antonio Costa

**Affiliations:** 1 Botanical Garden, Moscow State University, Moscow, Russia; 2 Laboratory of Biological Control of Insects, Faculdade de Ciências Biológicas e Ambientais, Universidade Federal da Grande Dourados, Dourados, Mato Grosso do Sul, Brazil; 3 Departamento de Ciências Biológicas, Universidade Estadual de Santa Cruz, Ilhéus, Bahia, Brazil

**Keywords:** Parasitoids, chromosomes, karyotypes, NORs, CMA_3_, DAPI

## Abstract

Chromosomes of three chalcid wasp species from Brazil, *Palmistichus elaeisis* Delvare et LaSalle, 1993, *Trichospilus diatraeae* Cherian et Margabandhu, 1942 (both belonging to the family Eulophidae) and *Trichogramma pretiosum* Riley, 1879 (Trichogrammatidae), were studied using chromosome morphometrics and base-specific fluorochrome staining. The present study confirmed that these species respectively have 2n = 12, 14 and 10. Chromomycin A_3_ / 4’, 6-diamidino-2-phenylindole (CMA_3_/DAPI) staining revealed a single CMA_3_-positive and DAPI-negative band within haploid karyotypes of both *Palmistichus elaeisis* and *Trichogramma pretiosum*. This CG-rich band clearly corresponds to the nucleolus organizing region (NOR). Moreover, analogous multiple telomeric bands found on all chromosomes of *Trichospilus diatraeae* may also represent NORs. Certain features of karyotype evolution of the phylogenetic lineage comprising both Eulophidae and Trichogrammatidae are discussed. The results obtained during the present study demonstrate the importance of chromosome research on tropical parasitoids that remain poorly known in this respect.

## Introduction

Parasitoid Hymenoptera are one of the most species-rich, taxonomically complicated and economically important insect groups (Godfray 1994), with their species number in the world fauna probably approaching one million (Quicke 1997). Despite rapid accumulation of karyotypic data, they are still available for just about 500 parasitoid species (Gokhman 2009). Moreover, chromosome sets of only a few members of this group coming from tropical regions are studied up to now (see e.g. Silva-Junior et al. 2000a), which substantially hampers our knowledge of worldwide patterns of karyotype structure and evolution in parasitoid Hymenoptera. Furthermore, research of this kind rarely goes beyond the chromosome numbers and some other results of conventional staining (Silva-Junior et al. 2000b, Santos et al. 2015), and when it does, it often reveals previously unknown karyotypic features (Carabajal Paladino et al. 2013). To promote better understanding of the above-mentioned patterns, we have recently studied chromosome sets of three polyphagous parasitoids from Brazil, i.e., *Palmistichus elaeisis* Delvare et LaSalle, 1993, *Trichospilus diatraeae* Cherian et Margabandhu, 1942 (both belong to the family Eulophidae) and *Trichogramma pretiosum* Riley, 1879 (Trichogrammatidae) using both routine (Giemsa) and base-specific fluorochrome staining.

## Material and methods

### Origin of parasitoids

Parasitoid strains were kept as lab stocks in the Laboratory of Biological Control of Insects of the Faculdade de Ciências Biológicas e Ambientais of the Universidade Federal da Grande Dourados, Dourados, Mato Grosso do Sul, Brazil, in climate-controlled chambers at 25 ± 1°C, 70 ± 10% relative humidity, and 14-hour photoperiod. All examined strains were maintained on lepidopteran hosts. Specifically, both studied members of the family Eulophidae and *Trichogramma pretiosum* were respectively bred on the pupae of *Diatraea saccharalis* (Fabricius, 1794) (Crambidae) and eggs of *Ephestia kuehniella* (Zeller, 1879) (Pyralidae). Parasitoids were identified by Marcelo Teixeira Tavares (Eulophidae) and Ranyse Barbosa Querino da Silva (Trichogrammatidae).

### Preparation of chromosomes

Chromosomal preparations were obtained from cerebral ganglia of parasitoid prepupae taken from dissected hosts generally following the protocol developed by Imai et al. (1988) with certain modifications (see Gokhman et al. 2016). Five to seven individuals of each species were studied. Ganglia were extracted from insects dissected in 0.5% hypotonic sodium citrate solution containing 0.005% colchicine. The extracted ganglia were then transferred to a fresh portion of hypotonic solution and incubated for 30 min at room temperature. The material was transferred onto a pre-cleaned microscope slide using a Pasteur pipette and then gently flushed with Fixative I (glacial acetic acid: absolute ethanol: distilled water 3:3:4). The tissues were disrupted using dissecting needles in an additional drop of Fixative I. Another drop of Fixative II (glacial acetic acid: absolute ethanol 1:1) was applied to the center of the area, and the more aqueous phase was blotted off the edges of the slide. The slides were then dried for approximately half an hour and stored at -20°C.

### Chromosome staining

For conventional staining, preparations were stained with freshly prepared 3% Giemsa solution in 0.05M Sørensen’s phosphate buffer (Na_2_HPO_4_ + KH_2_PO_4_, pH 6.8) for about 15 min. Fluorochrome staining with chromomycin A_3_ and 4’, 6-diamidino-2-phenylindole (CMA_3_/DAPI) was performed according to Schweizer (1976) with certain modifications by Guerra and Souza (2002). The slide was flooded with CMA_3_ staining solution (0.5 mg/ml in McIlvaine’s buffer), covered with a coverslip, and incubated at room temperature in the dark for 1 hour. The coverslip was then removed, and the slide was briefly rinsed with distilled water and air-dried. The slide was then flooded with DAPI solution (2 μg/ml in McIlvaine’s buffer), covered with a coverslip, and stained in the dark at room temperature for 30 min. The coverslip was then removed, and the slide was briefly rinsed with distilled water before being air-dried. The preparation was then mounted in Vectashield anti-fade medium (Vector Laboratories). The slide was stored in the dark prior to examination for a minimum of three days.

### Image acquisition and analysis

Metaphase plates were analyzed under an Olympus BX51 microscope. Images of chromosomes were taken with an Olympus DP72 camera using ImageProPlus software. To prepare illustrations, the resulting images were arranged and enhanced using Adobe Photoshop 8.0. The same software was also used for taking measurements from selected metaphase plates with good chromosome morphology. Statistical analysis was performed using STATISTICA 5.5. The chromosomes were classified following guidelines provided by Levan et al. (1964).

## Results

### *Palmistichus elaeisis*

**Figure 1.**
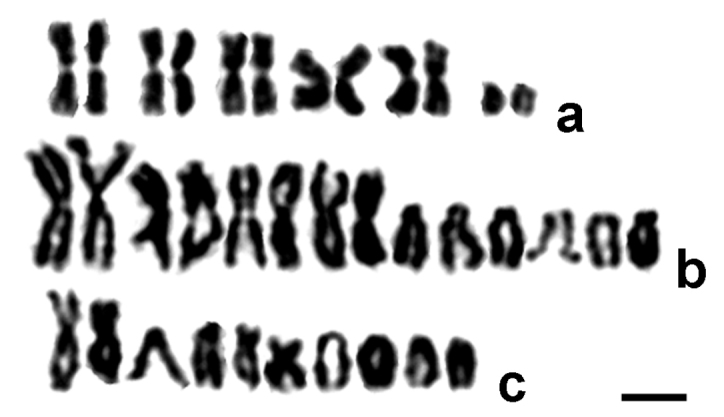
Diploid karyotypes of parasitoids. **a** *Palmistichus elaeisis* **b** *Trichospilus diatraeae* **c** *Trichogramma pretiosum*. Bar = 5 μm.

**Figure 2.**
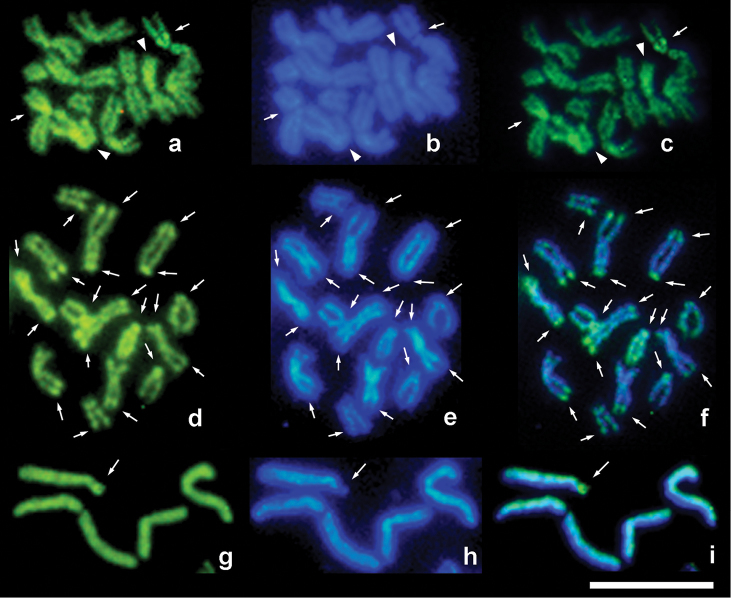
CMA_3_/DAPI-stained metaphase plates of parasitoids. **a–c** *Palmistichus elaeisis*, female **d–f** *Trichospilus diatraeae*, female **g–i** *Trichogramma pretiosum*, male **a, d, g** CMA_3_ staining **b, e, h** DAPI staining **c, f, i** merged CMA_3_/DAPI staining. Arrows and arrowheads respectively indicate CMA_3_-positive and DAPI-negative bands (NORs) as well as both CMA_3_- and DAPI-positive chromosome arms. Bar = 10 μm.

Five pairs of large metacentric chromosomes of similar size and a much smaller pair of acrocentrics were found in this species with 2n = 12 (Fig. 1a; Table 1). Fluorochrome staining of the diploid karyotype revealed a single pericentromeric CMA_3_-positive and DAPI-negative band on both homologous medium-sized metacentric chromosomes, probably the second longest ones (Fig. 2a–c). In addition, both CMA_3_- and DAPI-positive arms were visualized on smaller chromosomes of another metacentric pair.

### *Trichospilus diatraeae*

The karyotype of this species contains four pairs of large metacentrics of approximately the same size (the first pair is slightly longer than the remaining ones) and three considerably smaller pairs of acrocentric chromosomes; the chromosome number in *Trichospilus diatraeae* is thus 2n = 14 (Fig. 1b; Table 1). Surprisingly, fluorochrome staining visualized multiple telomeric CMA_3_-positive and DAPI-negative bands on virtually all chromosomes (Fig. 2d–f).

### *Trichogramma pretiosum*

The largest chromosome pair in the karyotype of this species is metacentric, whereas the remaining four pairs are clearly smaller; three of them are somewhat similar in size, and the fourth one is substantially shorter (2n = 10). Most these chromosomes are acrocentric (pairs no. 2, 4, and 5) except for the third pair of metacentric chromosomes which is more or less equal in length to the fourth pair of acrocentrics (Fig. 1c; Table 1). As in *Palmistichus elaeisis*, a single CMA_3_-positive and DAPI-negative band is revealed on chromosomes of *Trichogramma pretiosum* using fluorochrome staining (Fig. 2g–i). This pericentromeric band is localized on the largest acrocentric.

**Table 1.** Relative lengths (RL) and centromeric indices CI) of parasitoid chromosomes (mean ± SD). For each species, numbers of analyzed diploid metaphase plates are given in brackets.

**Chr. no.**	***Palmistichus elaeisis* (6)**		***Trichospilus diatraeae* (4)**		***Trichogramma pretiosum* (15)**	
	**RL**	**CI**	**RL**	**CI**	**RL**	**CI**
1	20.44 ± 0.78	48.43 ± 2.42	20.28 ± 0.72	44.28 ± 4.08	25.94 ± 1.48	42.32 ± 2.92
2	19.47 ± 0.42	47.57 ± 1.67	18.38 ± 0.72	45.17 ± 3.72	20.55 ± 0.83	0
3	18.68 ± 0.30	47.64 ± 1.86	16.75 ± 0.91	44.44 ± 3.75	18.97 ± 0.78	44.89 ± 2.73
4	17.88 ± 0.32	47.61 ± 1.59	14.88 ± 0.68	44.51 ± 4.45	18.66 ± 0.84	0
5	16.98 ± 0.45	46.61 ± 1.97	11.20 ± 0.72	0	15.88 ± 0.95	0
6	6.55 ± 0.39	0	10.18 ± 0.66	0	-	-
7	-	-	8.33 ± 0.66	0	-	-

## Discussion

Chromosome sets of *Palmistichus elaeisis* and *Trichospilus diatraeae* were first studied by Silva-Junior et al. (2000a) who respectively found 2n = 12 and 14 in these species. We therefore confirm these results. The karyotype structure of *Palmistichus elaeisis* is similar to that of many other members of the subfamily Tetrastichinae. Moreover, this structure is apparently the commonest in the family Eulophidae in general (Gebiola and Bernardo 2008; Gokhman 2009, Gokhman and Gumovsky 2009, Bolsheva et al. 2012, Gokhman et al. 2014b) which is, in turn, one of the best karyotypically studied chalcid families (see e.g. Gokhman 2009, Gebiola et al. 2012). The chromosome set of *Trichospilus diatraeae* (Eulophinae) is also structurally similar to those of most members of the family Eulophidae, except for the two additional pairs of acrocentrics instead of a larger pair of metacentric chromosomes. Silva-Junior et al. (2000a) therefore hypothesized that this particular feature had probably resulted from a centric fission, and we agree with this assumption. Interestingly, another tropical/subtropical parasitoid, *Chelonus insularis* Cresson, 1865 (Braconidae), also shows n = 7 (Silva-Junior et al. 2000b), as opposed to n = 6 in all other studied species of temperate Cheloninae (Gokhman 2009), and this is also interpreted as a case of centric fission.

The chromosome set of *Trichogramma pretiosum* was previously studied by Hung (1982) together with three other members of this genus. All these parasitoids had n = 5, and this author also postulated highly similar karyotype structure of those species in terms of relative chromosome lengths and centromeric indices. Specifically, Hung (1982) reported that haploid chromosome sets of those parasitoids harbored a larger submetacentric, a smaller metacentric and three smallest acrocentric chromosomes of the same size (one of them with an obvious shorter arm), with approximate RLs of 26.5, 21 and 3 × 17.5 percent respectively (see Fig. 7 in Hung 1982). However, images of metaphase plates provided by this author suggest that chromosomal morphology of certain *Trichogramma* Westwood, 1833 species can substantially differ from the above-cited pattern. It is therefore not surprising that we have found a slightly different karyotype structure in *Trichogramma pretiosum* as well (Table 1). In particular, the first pair of chromosomes is clearly metacentric. Moreover, another medium-sized pair of metacentric chromosomes is the third/fourth longest one within the karyotype (Fig. 1c), not the second, as it was shown in Hung (1982). In addition, no acrocentric chromosome carries a clearly visible shorter arm, and all acrocentric pairs seemingly differ in their lengths in this species.

Staining of *Palmistichus elaeisis* karyotype with certain base-specific fluorochromes was apparently performed for the first time by Silva-Junior et al. (2000a). However, these authors neither published images of stained chromosomes nor gave a detailed description of the results obtained. Within previously studied parasitoid karyotypes, GC-rich, i.e. CMA_3_-positive (and DAPI-negative) regions corresponded only to NORs (Bolsheva et al. 2012, Gokhman et al. 2016). Our results therefore demonstrate presence of the single NOR in the chromosome set of *Palmistichus elaeisis*. As far as other members of the subfamily Tetrastichinae are concerned, AgNOR-banding also visualized the only NOR on a larger metacentric chromosome (perhaps the second longest one) in the karyotype of *Melittobia australica* Girault, 1912 (Maffei et al. 2001). In addition, CMA_3_/DAPI staining revealed a single NOR in the chromosome set of another member of the same genus, *Melittobia hawaiiensis* Perkins, 1907 (Silva-Junior et al. 2000a). However, NORs are localized on the smallest acrocentrics within karyotypes of some other Eulophidae, i.e. two *Entedon* Dalman, 1820 (Entedoninae) and several *Pnigalio* Schrank, 1802 (Eulophinae) species (Bernardo et al. 2008, Bolsheva et al. 2012, Gebiola et al. 2012). On the other hand, occurrence of multiple telomeric CMA_3_-positive bands found in the karyotype of *Trichospilus diatraeae* is apparently derived and quite unusual for parasitoids, but is known for aculeate wasps (Menezes et al. 2011) and bees (Duarte et al. 2009). In the latter study, most CMA_3_-positive bands are attributed to NORs, and this is probably true for *Trichospilus diatraeae* as well. As for both CMA_3_- and DAPI-positive chromosome arms found in *Palmistichus elaeisis*, segments of that kind detected in other Hymenoptera are usually heterochromatic (see e.g. Menezes et al. 2011). Interestingly, fully heterochromatic chromosome arms in parasitic wasps were also revealed, for example, on a particular pair of metacentric chromosomes of three species of the genus *Nasonia* Ashmead, 1904 (Gokhman and Westendorff 2000).

Karyotypes of *Trichogramma* species have never been examined using base-specific fluorochromes. Nevertheless, NORs in this genus were previously studied by van Vugt et al. (2005, 2009) who examined the chromosome set of *Trichogramma kaykai* Pinto et Stouthamer, 1997 using FISH with a 45S rDNA probe. In essence, two NORs were detected within the karyotype of this species (a third one was visualized on a particular B chromosome). However, we observed a single NOR on the chromosomes of *Trichogramma pretiosum*, indicating that the genus *Trichogramma* can harbor different species which karyotypes have either one or two NORs. Variation of this kind was earlier detected within the chalcid genus *Eurytoma* Illiger, 1807 (Eurytomidae; Gokhman et al. 2014a).

The chalcid families Eulophidae and Trichogrammatidae belong to the same phylogenetic lineage that also includes Aphelinidae and a few smaller groups of which the chromosomes are as yet unknown (Heraty et al. 2013). The present study can therefore add to our knowledge of the karyotype evolution within this lineage. In particular, both Eulophidae and Aphelinidae appear to have a single NOR per haploid karyotype (Baldanza et al. 1999, Baldanza and Giorgini 2001, Giorgini and Baldanza 2004, Bernardo et al. 2008, Bolsheva et al. 2012, Gebiola et al. 2012, Gokhman et al. 2017). On the contrary, haploid chromosome sets of various *Trichogramma* species studied in this respect can carry either one or two rDNA sites (van Vugt 2005, 2009 and the present paper). However, since higher numbers of rDNA clusters are probably ancestral for the superfamily Chalcidoidea as well as for parasitic wasps in general (Gokhman et al. 2014a), presence of a single NOR in all these groups may represent a derived state.

In conclusion, we would like to stress the importance of studying chromosome sets of tropical parasitic wasps for our understanding of the karyotype structure and evolution in parasitoid Hymenoptera in general. For example, the chromosome set of *Trichospilus diatraeae* demonstrates deviating karyotypic features in terms of both Giemsa and CMA_3_/DAPI staining. In particular, the presence of multiple CMA_3_-positive telomeric bands on all chromosomes of this species was not previously detected in any other parasitoid. Moreover, this study reveals for the first time considerable karyotypic differences within the speciose and practically important genus *Trichogramma*, again in terms of both chromosome morphometrics and the number of NORs, thus potentially contributing to our knowledge of its taxonomy and phylogeny. Our data therefore confirm that further research of parasitoid Hymenoptera from various geographical regions will undoubtedly demonstrate more variation in the overall karyotype structure and distribution of different chromosome segments in this group.
